# Trust in Health Care Providers, Anxiety, Knowledge, Adherence to Guidelines, and Mental Healthcare Needs Regarding the COVID-19 Pandemic

**DOI:** 10.1177/21582440231179125

**Published:** 2023-06-16

**Authors:** Gizell Green, Riki Tesler

**Affiliations:** 1 Ariel University, Israel

**Keywords:** Trust in healthcare, anxiety about COVID-19, knowledge about COVID-19, adherence to guidelines, mental healthcare needs

## Abstract

The mechanisms of the connections among anxiety, mental healthcare needs, and adherence to the COVID-19 pandemic guidelines are unknown. The study aims to explore model assumptions: (H1) Anxiety about COVID-19 will influence the perception of mental health needs via knowledge about COVID-19 as a mediator. (H2) Anxiety will influence adherence to guidelines via knowledge about COVID-19 as a mediator. (H3) Trust in health care will positively influence adherence to guidelines. We conducted a cross-sectional design study with a convenience sample. Participants consisted of 547 people across Israel. The questionnaire included trust in health care, anxiety, knowledge, adherence to guidelines, and mental health care needs regarding COVID-19 variables. Path analysis revealed knowledge about COVID-19 as partly mediating anxiety and mental healthcare needs during the pandemic, as well as partly mediating anxiety and adherence to the pandemic guidelines. Moreover, we found that trust in healthcare affects adherence to the pandemic guidelines. Therefore, it is important to design an intervention program for the public providing accessible, reliable information about the pandemic, including, and emphasizing mental healthcare needs and rationale of adherence to the guidelines.

## Introduction

Since December 2019, a growing number of cases of the COVID-19 virus have been diagnosed. The virus was initially discovered in Wuhan, a large city in China (Li et al., 2020), and quickly spread worldwide, including in Israel (Li et al., 2020; Schwartz et al., 2020; Situation Report-65 HIGHLIGHTS, n.d). On January 30, 2020, the world health organization (WHO) conducted an emergency meeting and declared the worldwide COVID-19 outbreak, later naming it a pandemic (WHO, 2020). The COVID-19 virus expands through the respiratory tract, with immediate contact triggering a severe respiratory disease (Guo et al., 2020). Virus symptoms vary from no symptoms at all to serious health problems, such as acute respiratory distress syndrome, body failure, and eventually death (Grein, 2020). Therapy for COVID-19 illness in the intensive care setting may be vital (Arabi et al., 2020). The COVID-19 pandemic has affected the quality of life of individuals; with the physical distancing recommended strongly influencing people’s lives (Di Renzo et al., 2020). During this time, an elevated level of anxiety was identified (Moghanibashi-mansourieh, 2020).

In the first wave in Israel, there were 500 to 700 brand new proved incidents a day. Furthermore, Israel had a mortality rate of fewer than 300 individuals, representing 33 diseases per million, and a fatality rate of 1.67%, lesser than in than majority of European countries (Birenbaum-Carmeli & Chassida, 2021). However, in June 2020, the volume of new proven incidents of COVID-19 started increasing, to 4,000 to 6,000 new incidents per day in the middle of September. At that moment, with a total of approximately 180,000 approved incidents, 50,000 of which were active, Israel turns into a very infected country. This happened in the second wave (Birenbaum-Carmeli & Chassida, 2021).

The unexpected pandemic can cause psychological distress, such as anxiety, depression, and other mental disorders (Huang & Zhao, 2020; Roy, Tripathy, Kumar, & Sharma, 2020; Y. Wang et al., 2021). Research has found that more than 80% of participants demonstrated high anxiety levels related to the pandemic (Roy, Tripathy, Kumar, & Sharma, 2020). This anxiety is a global phenomenon, with an impact on every individual in almost every aspect of life (Roy, Tripathy, Kumar, & Sharma, 2020). Another study that investigated participants’ knowledge about Covid-19 found that the information on the increase in the number of recovered individuals was significantly associated with a low anxiety score (Wang et al., 2020). Furthermore, other study argue that knowledge, attitudes, anxiety, and perceptions of mental health needs among the subjects with easy and difficult internet access may be different (Mundakir et al., 2020).

Therefore, consistent with cautions from mental health professionals regarding an “echo pandemic”—the spread of mental health problems, such as anxiety, depression, and other mood disorders, likely to be high (Dozois, 2021), mental healthcare needs have become crucial. However, the connection between anxiety and mental healthcare needs can be complex and is not unrelated to, other factors, as well as may involve mediator variables, such as knowledge about COVID-19.

During the 2003 SARS-CoV epidemic, researchers found that some levels of anxiety were associated with participants’ higher adherence and preventive actions (Leung et al., 2003). They adhered to the guidelines for preventing infection by the disease. COVID-19s’ specific precautionary measures include avoidance of sharing tools (e.g., chopsticks), hand sanitation, and wearing masks (Wang et al., 2020). In the framework of a pandemic unrecognized, anxiety might prompt attentive guideline adherence (Krupić et al., 2021).

However, the connection between anxiety and COVID-19 guidelines adherence may be influenced by other factors, and as it is not unconnected to other issues, it may involve a mediator variable, such as knowledge about COVID-19. As knowledge levels are linked with psychological elements, such as panic, among most individuals, this may affect their views and behavior toward COVID-19 (Zhong et al., 2020).

In addition, the research found that public confidence and trust in the health care system are positively related to adherence to the pandemic guidelines (Chan et al., 2020).

Such model involved variables mentioned above can provide infrastructure necessary to address health system for efficient, and equitable delivery of mental health-care delivery. Furthermore, after extensive literature research, no studies were found that examined innovative model gathering variables such as trust in health care providers, adherence to COVID-19 guidelines, Anxiety-related to COVID-19, perceived mental healthcare needs, and knowledge about COVID-19. Furthermore, this model strives to find knowledge about COVID-19 as a mediator function in this model.

### Research Questions and Hypothesis According to the Model

1. Does anxiety about COVID- 19 will influence the perception of mental health needs via knowledge about COVID-19 as a mediator?

Therefore, we hypothesized:

(H1) Anxiety about COVID- 19 will influence the perception of mental health needs via knowledge about COVID-19 as a mediator.
(H1a) Anxiety will influence mental health needs.
(H1a) Anxiety will influence knowledge about COVID-19.
(H1b) Knowledge about COVID-19 will influence the perception of mental health needs during the COVID-19 epidemic.
2. Does anxiety will influence adherence to the guidelines via knowledge about COVID-19 as a mediator?

Therefore, we hypothesize:

(H2) Anxiety will influence adherence to the guidelines via knowledge about COVID-19 as a mediator.
(H2a) Anxiety will influence adherence to COVID-19 guidelines.
(H2b) Knowledge will influence adherence to COVID-19 guidelines.
3. Does trust in healthcare will positively influence adherence to COVID-19 guidelines?

Accordingly, we hypothesize:

(H3) Trust in healthcare will positively influence adherence to COVID-19 guidelines.

## Materials and Methods

### Study Design

We conducted a cross sectional-design study with a convenience sample. The data was collected from May 2020 to August 2020.

### Participants and Procedure

Participants included 547 people from all across Israel. As of early May 2020, the number of total COVID-19 cases in Israel was 16,101, with 2,225 deceased (Worldometer, 2021). The answers to the questionnaire were collected via iPanel (https://www.ipanel.co.il/en/), an online sampling service allowing the fast collection of answers from a representative sample based on population sociodemographic components, such as gender, age, and education. This is the largest panel survey in Israel, embracing high-quality research codes from the European Society for Opinion and Marketing Research (Bodas & Peleg, 2020; Bodas et al., 2017). The data was collected from May 2020 to August 2020.

### Tools

The questionnaire was composed of six parts:

**Part A:Trust in healthcare** of providers was assessed using the trust in healthcare scale, a 17-item scale consisting of three subscales: trust in healthcare providers, payers (insurance), and trust in healthcare institutions. This study used the trust in healthcare providers subscales and for this internal validity was .92 (Cronbach’s α). We choose to use only the trust in healthcare providers subscale which (contains 10 items) since the other subscales didn’t fit Israel’s healthcare system. All items were scored on a 5-point Likert scale, ranging from 5 (strongly agree) to 1 (strongly disagree). Example of item: “Healthcare institutions provide the highest quality in medical care”. “My health care provider offers me the highest quality in medical care” (Egede & Ellis, 2008).

**Part B:The attitude toward COVID-19**, showed peoples’ adherence to the COVID-19 guidelines. It contained five items rated on a 5-point Likert scale, ranging from 5 (strongly agree) to 1 (strongly disagree), with a Cronbach’s of .52. After deleting two items Cronbach’s was .65. Example of item: “Maintaining distance between people is essential to eradicate the spread of the coronavirus” (Roy, Tripathy, Kumar, & Sharma, 2020). Since internal validity of the scale was still insufficient (below 0.7), exploratory factor analysis was conducted, due to the deletion of the two items we performed expletory factor analysis (EFA) for the remaining item. See Table 1.

**Table 1.** Exploratory Factor Analysis of Attitude Towards COVID-19 Showed Peoples’ Adherence to the COVID-19 Guidelines (Roy, Tripathy, Kumar, & Sharma, 2020).

Scale items	Fac1
Washing hands frequently can lower the risk of coronavirus infection	0.81
Maintaining distance between people is essential to eradicating the spread of the coronavirus.	0.83
If I have a fever or cough, I likely isolate myself	0.68
Eigenvalue	1.81

Fac1 corresponds to the factor’s “adherence to the COVID-19 guidelines.”

The factor analysis yielded one factor and all items’ coefficients were above .68, therefore all three items remained. Additionally, construct validity was performed (convergent and discriminant validity) for matrix correlation, see Table 2.

**Table 2.** Matrix Correlation of Adherence to COVID-19 Guidelines (Roy, Tripathy, Kumar, & Sharma, 2020) and COVID-19 Skepticism and Concerns.

Variables	Washing hands frequently can lower the risk of coronavirus infection	If I have a fever or cough, I likely isolate myself	Maintaining distance between people is essential to eradicating the spread of the coronavirus	COVID-19 Skepticism	Covid-19 concerns
Washing hands frequently can lower the risk of coronavirus infection	1	0.39**	0.50**	−0.37**	0.48**
If I have a fever or cough, I likely isolate myself		1	0.75**	−0.60**	0.53**
Maintaining distance between people is essential to eradicating the spread of the coronavirus			1	−0.55**	0.49**
COVID-19 Skepticism				1	−0.40**
Covid-19 concerns					1

Notes: *p < 0.05; **p < 0.00.

Table 2 shows that convergent validity is positively related to the three items of adherence to the COVID-19 guidelines and Covid-19 concerns scale (0.48 < *r* < 0.53) (Chou et al., 2005). In addition, discriminant validity is positively related to the three items of adherence to the COVID-19 guidelines and COVID-19 the skepticism scale (−0.60 < *r* < −0.37) (Latkin et al., 2021).

**Part C: Anxiety related to the COVID-19 disease** had 18 items rated on a 5-point Likert scale (never (1), occasionally (2), sometimes (3), often (4), and always (5), with a Cronbach’s of .89. Example of item: “In the past week, how often do you feel worried about yourself and your loved ones regarding the spread of the new COVID-19 viral infection?” (Roy, Tripathy, Kumar, & Sharma, 2020).

**Part D: Perceived mental healthcare needs** were assessed by four items, where the participant was asked to answer either yes = 2, neutral = 1, or no = 0, for each question. Example of item: “Do you think it would be beneficial if mental health professionals would help people deal with the current COVID-19 pandemic situation?” The score consisted of the sum of individual answers, with higher total and subscale scores representing a greater mental healthcare need perceiving (Roy, Tripathy, Kumar, & Sharma, 2020).

**Part E: Knowledge about COVID-19** included six multiple-choice questions examining knowledge about COVID-19. The score consisted of the sum of individual correct answers so that higher total scores represent greater knowledge about COVID-19. Example of item: “Signs of infection with the coronavirus are:” (Roy, Tripathy, Kumar, & Sharma, 2020).

**Part F:Background characteristics** included information regarding participants’ background, such as age and gender.

### Statistical Analysis

All statistical analyses were performed using SPSS 25.0. In addition, we used the Second, R software (version 3.5) developed by R Core Team (Ripley, 2001). We conducted a series of regression analyses for examining the mediation model. It was conducted to establish path relationships among the variables, and several SEM model indices examined Goodness of Fit Index (GFI), Root Mean Square Error of Approximation (RMSEA), Tucker Lewis Index (TLI), Normed Fit Index (NFI), Standardized Root Mean Squared Residual (SRMR), and χ^2^, *p* > .05 (see Table 3).

**Table 3.** Background Characteristics of the Study Population.

Background characteristic	*N* = 547	
	N	%
Gender		
Male	155	28
Female	388	71
Status		
Single	193	35
Married/partnership	315	58
Divorced	29	5
Widowed	3	1
Religion		
Jewish	528	96
Muslim	6	1
Other	7	1
Education		
High school	127	23
Diploma	112	21
Bachelor’s degree	196	36
Master’s degree	94	17
PhD	12	2
Financial status		
Above-average wage	94	17
Average wage	83	15
Below average wage	358	66

SD = standard deviation.

Discrepancies in n’s due to missing values

### Ethical Considerations

The University Institutional Review Board (IRB) approved this study. Participants were recruited voluntarily and informed regarding the study aims. They also signed an informed consent form before responding to the survey. The participants were guaranteed that they had the right to leave the study at any time, that their answers would be confidential, and that the surveys would be analyzed anonymously.

## Results

The mean age of the participants was 33.59 (*SD* = 11.34). For frequency and percentage of background characteristics, see Table 3.

Table 3 shows that most of the participants were female, married/in a partnership, Jewish, had bachelor’s degrees, and earned a below-average wage.

We conducted a Pearson’s correlation coefficient to examine the potential connections among the study variables see, Table 4.

**Table 4.** Pearson Correlations Among Variables.

Variables	Trust in health care	Adherence to COVID-19 guidelines	Anxiety-related to COVID-19	Perceived mental healthcare needs	Knowledge about COVID-19
Trust in health care	1	0.21**	0.12**	0.05	0.10*
Adherence to COVID-19 guidelines		1	0.37**	0.25**	0.35**
Anxiety-related to COVID-19			1	0.16**	0.19**
Perceived mental healthcare needs				1	0.30**
Knowledge about COVID-19					1

* *p* < .05. **p < .00.

Table 4 shows significant correlations among almost all variables, except the variables “trust in healthcare” and ‘perceived mental healthcare needs.

For the model fit indices and structural equation model of the research, see Table 5.

**Table 5.** Model Fit Indices.

The goodness of fit measures of the SEM	Parameter estimates	Minimum cutoff	Suggested by
Adjusted Goodness of Fit Index (GFI) Root Mean Square Error of Approximation (RMSEA) Parameter	0.99	>0.80	Gefen et al. (2000)
Tucker Lewis Index (TLI)	0.96	>0.90	Hu & Bentler (1999)
Normed Fit Index (NFI)	0.98		
Comparative Fit Index (CFI)	0.99	>0.90	Hair et al., (2010)
Standardized Root Mean Squared Residual (SRMR)	0.02		
Root Mean Square Error of Approximation (RMSEA)	0.04	<0.07	Steiger (2007)

**Figure 1.**
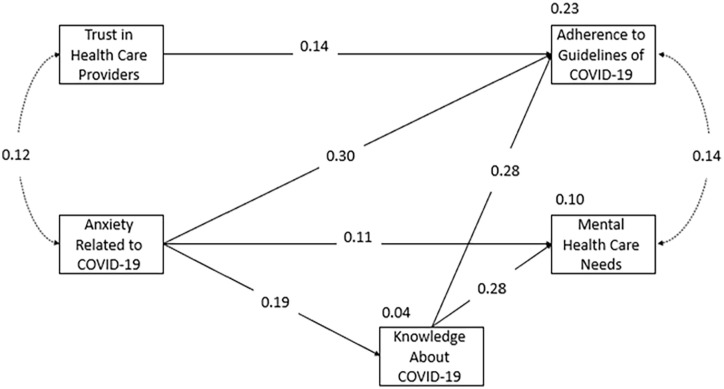
A path diagram showing the structural equation modeling analysis used to determine the paths among variables.

Table 5 shows that the model fit indices exhibited an acceptable model fit, but the results were not significant (χ^2^ = 3.89, *df* = 2, *p* = .142). See Figure 1.

Figure 1 shows that trust in healthcare has a significant direct effect on adherence to COVID-19 guidelines (β = .14). In addition, anxiety is directly related to adherence to COVID-19 guidelines, perceived mental healthcare needs, and knowledge about COVID-19 (β = .30), (β = .11), and (β = .19) respectively. Finally, knowledge about COVID-19 affects adherence to COVID-19 guidelines and perceived mental healthcare needs, (β = .28) and (β = .28) respectively.

In an effort to further explore the research model, we performed mediation analyses for the exposure of the direct and indirect effects of adherence to COVID-19 guidelines and perceived mental healthcare needs thorough knowledge about COVID-19, see Table 6.

**Table 6.** Mediation analyses.

Path	Effect	SE	*p*	95% CI
Anxiety-related to COVID-19 (*X*) → Knowledge (*m*) → Mental healthcare needs (*Y*)				
Total effect of *X* on *Y*	0.16	0.08	.00	0.16–0.51
Direct effect of *X* on *Y*	0.22	0.08	.01	0.05–0.39
Indirect effect of *X* on *Y*	0.11	0.03	.00	0.06–0.17
*X* → *M*	0.29	0.06	.00	0.17–0.42
*M* → *Y*	0.39	0.06	.00	0.28–0.50
Anxiety-related to COVID-19 (*X*) → Knowledge (*m*) → Adherence to the guidelines of COVID-19 (*Y*)				
Total effect of *X* on *Y*	0.37	0.04	.00	0.28–0.43
Direct effect of *X* on *Y*	0.29	0.04	.00	0.22–0.36
Indirect effect of *X* on *Y*	0.05	0.01	.00	0.03–0.08
*X* → *M*	0.29	0.06	.00	0.17–0.42
*M* → *Y*	0.18	0.02	.00	0.13–0.23

SE = standard error; CI = confidence interval.

Table 6 shows that mediation analysis has indicated that knowledge about COVID-19 partially mediated the effects of the anxiety related to COVID-19 and mental healthcare needs. Moreover, knowledge about COVID-19 partially mediated the effects of anxiety related to COVID-19 and adherence to COVID-19 guidelines.

## Discussion

As the mechanisms for the connections among anxiety, mental healthcare needs, and adherence to COVID-19 guidelines during the pandemic are unknown, this study examined whether knowledge about COVID-19 as a mediator was able to explain this relationship.

This cross-sectional study was the first to investigate the role of knowledge about COVID-19 as a dual mediator between anxiety and adherence to COVID-19 guidelines and perceived mental healthcare needs. Moreover, this study also examined the effect of trust in healthcare on adherence to COVID-19 guidelines. The (first) mediation analysis found that knowledge about COVID-19 partly mediated anxiety and mental healthcare needs during the pandemic. Similarly, it was also found that knowledge about COVID-19 partly mediated (second) between anxiety and adherence to COVID-19 guidelines. Moreover, it found that trust in healthcare affected adherence to COVID-19 guidelines.

First, was found that knowledge about COVID-19 partly mediated between anxiety and mental health needs. After a broad review of the contemporary research literature, we did not find a study that performed an in-depth analysis for detecting those variable relationships altogether. However, when we examined the relationships separably within this mediation model, the path (H1a) where anxiety increased mental health needs was found to be significant. This is consistent with other findings which show that during this pandemic, anxiety is likely to fit mental health needs, together with other mood disorders (Dozois, 2021; Roy, Tripathy, Kumar, & Sharma, 2020).

Another path (H1b), where anxiety increased the level of knowledge about COVID-19, was also found to be significant. The research literature demonstrated variety of results. This is consistent with another study which found that higher levels of anxiety are related to students who spend more than 1 hr per day looking for information on COVID-19 (Kecojevic et al., 2020) to enhance their knowledge. However, it is not consistent with another previous study conducted among health professionals, which demonstrated a connection between small levels of anxiety and good quality of knowledge and awareness of health (26) and another study found that the information on the increase in the number of recovered individuals related to decreased anxiety emotion (Wang et al., 2020). This may be related to the type of knowledge that participants are interested in.

The last path (H1c), knowledge about COVID-19, increased the perception of mental health needs during the pandemic. The study found that the more in-depth and inclusive the level of knowledge about negative and physical mental consequences (Huang & Zhao, 2020; Roy, Tripathy, Kumar, & Sharma, 2020), the more importance will be given to mental health needs during the pandemic. Furthermore, other study argue that knowledge, anxiety, and perceptions of mental health needs are reliant on easy or difficult internet entry and may be different (Mundakir et al., 2020).

Second, we found that knowledge about COVID-19 is partly mediated between anxiety and adherence to COVID-19 guidelines. Here, too, after an extensive search of the research literature, we did not find a study that examined this modal analysis. However, the study examined the relationships within this mediation model, the path (H2a) of anxiety increasing COVID-19 guidelines adherence, was found significant. This is consistent with other findings showing that during this pandemic, anxiety may also be related to individuals’ knowledge, attitudes, and practices toward COVID-19 (Al-Hanawi et al., 2020; Lee et al., 2006). Another study found that anxiety and fear regarding COVID-19 significantly positively predicted guideline adherence (Segal et al., 2020). Within the context of an unfamiliar highly infected virus, anxiety might induce compliance and adherence to the guidelines (Krupić et al., 2021).

Another path (H2b) found that knowledge about COVID-19 increased adherence to COVID-19 guidelines. These findings are similar to another study that found that knowledge is important in molding individuals’ behavior and practices, particularly throughout an outbreak of a disease (Abdulkareem et al., 2020). People’s knowledge and attitudes are expected to influence the extent of maintaining personal protective practices (Roy, Tripathy, Kar, et al., 2020).

Third, it was found that healthcare trust positively affects adherence to COVID-19 guidelines (H3). These findings are similar to the general assumption that a lack of trust in health organizations damages compliance (Torgler et al., 2011), which can, in turn, lead to reduced compliance with policies enacted by the state (Fai et al., 2020).

## Conclusions

The study found that knowledge about COVID-19 partly mediated anxiety and mental healthcare needs during the pandemic, as well as partly mediated anxiety and adherence to COVID-19 guidelines. Moreover, we found that trust in healthcare affects adherence to COVID-19 guidelines. It is important to design intervention programs for the public, providing accessible, reliable information about the pandemic, including, and emphasizing mental healthcare needs and rationale of adherence to the COVID-19 guidelines.

### Practical Implications

The novelty of the model’s findings in this study highlights the importance of variables such as trust in health care providers’ anxiety related to COVID-19 and knowledge about COVID-19, effecting/mediating variables adherence to guidelines of COVID-19, and mental health care needs Accordingly to the research model anxiety related to COVID-19 is an important factor and knowledge about COVID-19 as a dual mediator. Thus, that can provide infrastructure to address health system weaknesses by disseminating good practices that can result in efficient, and equitable delivery of mental health-care delivery. Thus, based that the COVID-19 pandemic could be an opportunity to improve mental health services, they need to implement service changes to support accessibility and continuity of care and reinforced population well-being. Services also should guarantee they are communicating important information in a well-defined and available manner with the population, regarding service delivery, contamination symptoms, government guidelines, and more.

### Limitations and Recommendations for Future Research

There are a few limitations to the study. The novelty of the model’s findings in this study highlights the importance of variables such as trust in health care providers’ anxiety related to COVID-19 and knowledge about COVID-19, effecting/mediating variables adherence to guidelines of COVID-19, and mental health care needs. Therefore, additional studies are required to validate these important findings for an optimal prospective guideline to be performed in similar future unexpected events. Second, the study was performed via a convenience sample in one state, Israel, and has reduced research sample representativeness. Thus, it is recommended to explore and replicate this study in a larger sample and several countries. Another limitation is the implementation of a single tool (questionnaire) in this study. Therefore, further research should compose several tools, such as interviews, which may be useful for detecting complex perceptions of the participants.
